# The ExoGAN generative AI framework enables extracellular vesicle-based immunotherapy

**DOI:** 10.21203/rs.3.rs-6915262/v1

**Published:** 2025-06-29

**Authors:** Kiley Graim, Zachary Greenberg, Tina Salehi Torabi, Jiayu Huang, Franco Krepel, James Cahill, David Ostrov, Mei He

**Affiliations:** University of Florida

## Abstract

Neoantigens, specifically tumor antigenic peptides presented by MHC-I, are essential for activating cytolytic CD8+ T cells via T Cell Receptor (TCR) recognition. Neoantigens have drawn growing attention for developing novel cancer immunotherapy approaches. However, current cancer immunotherapy suffers from low and unpredictable response rates due to the heterogeneity and unknown TCR binding properties among human patients. To address this issue, here we introduce the ExoGAN generative AI framework to guide the design of novel tumor antigenic peptides. ExoGAN is a generative adversarial network (GAN) that integrates HLA physiochemistry feature engineering with sequence-level data, used here to design new HLA-A*02:01-targeting peptides. We apply ExoGAN to design human-based neoantigens that bind to specific MHC-Is (HLAs) with improved TCR recognition and T cell activation, training the model on the largest IEDB dataset of HLA peptides. To deliver these ExoGAN-designed peptides to cells for effective T cell activation, we incorporate a membrane surface molecular engineering approach to fully decorate extracellular vesicles (EVs) with ExoGAN-designed peptides in MHC complex proteins. EVs have been recognized as essential immunity mediators, which carry costimulators with antigenic presentation for leading the emerging and advanced cancer immunotherapy, making them ideal for peptide delivery. Computational and experimental validation shown here, demonstrate that the ExoGAN-generated peptide carrying EVs consistently express antigenic presentation to T cell TCRs, resulting in precisely controlled and programmable EV agents for anti-tumor immunity activation. This greater coverage of effective antigens advances mechanistic understanding of neoantigen functionality, despite inconsistent TCR signaling across human patients. Combined with EV immunity activation, our ExoGAN framework is deployable for clinical translation and enabling precision cancer immunotherapy. The increased scientific understanding of neoantigen diversity across tumors, discovered by ExoGAN, will also accelerate the development of new functional immunotherapy agents in activating patient-specific TCRs.

## Introduction

Neoantigens are tumor-associated peptides that bind the Major Histocompatibility Complex (MHC-I), then functionally activate CD8+ T cells through molecular interaction with a known T Cell Receptor (TCR). Deep learning and other artificial intelligence (AI) approaches have become popular for identifying potential neoantigens^1-9^ in cancer immunotherapy research, for example as cancer vaccines^10,11^. Many existing AI models predict the likelihood that a given peptide is a neoantigen based on predicted MHC-I binding affinity ^5-7,12-19^. Most utilize gold-standard datasets from the Immune Epitope Database (IEDB) as training data to screen peptides for neoantigen characteristics^6,7,14-19^ or for *de novo* neoantigen^5,12,13^. For both approaches, AI models have primarily focused on leveraging known neoantigen sequences or the sequences plus their respective MHC-I molecule binding target. ^5,12,13,20^

Current neoantigen genAI frameworks operate by using agents^12,13^, generative adversarial networks (GANs)^5^, and diffusion^20^ models. Agent-based genAI models, for example UltraMutate^12^ and PepPPO^13^, use enhanced policy gradients to optimize residue mutations in generated peptides to predict binding affinity. Most of these pipelines assume that immunogenic peptides are strong binding peptides and the generated sequences are based on sequence-level similarity with training data rather than physiochemistry similarities. Overfitting the training data could be a potential issue, making the experimental validation inconsistent^21-23^. On the other hand, the training data is biased towards certain sequence motifs, which leads to being skewed with current models due to such dataset challenge ^21-23^. Databases such as the Protein Data Bank (PDB) could provide the large-scale training data that genAI methods require for improved model training. However, PDB crystal structures do not contain information detailing the physiological conditions in which neoantigens are found in biological contexts. While it is possible to compute this information, doing so across the entirety of PDB (234,785 experimental and 1,068,577 computed structures as of April 16, 2025) requires significant compute resources and domain expertise, rendering the creation of this information at a large enough scale for genAI model training a gargantuan task.

Fortunately, previous works in determining chemical processes have provided methods to capture this biological context through quantum and molecular mechanics (QM/MM)^24^ calculations. QM/MM methods^25^, awarded the 2013 Nobel Chemistry prize, provide the means to determine and verify chemical reactivity in biomolecular systems. Using these approaches on peptides elucidates the contribution of specific electrostatic interactions available to peptides and their specific residues^26-28^ interacting with proteins, which can be applied to compute a peptide’s electronic structure, in turn, determining a peptide’s chemical potential including solvation-free energy, molecular conformation, dipole moment, solvent-accessible area and volume, and rotational movement vectors. Further physiochemical theory may be applied post QM/MM to determine lower-level parameters including molecular stiffness, entropy, and diffusion in targeted physiological conditions. Only one report in the literature exists for MHC-I peptide binding calculations^28^, which evaluated the impact of water and individual residue contributions to binding MHC-I. In that publication, Li et al reported explainable physiochemistry, including Gibb’s free energy for the peptides, and pinpointed residues interacting with a human MHC-I, HLA*02:01, with their results correlated well with the experimentally known binding affiity. Many additional QM/MM calculations could be used to decipher the underlying rules to neoantigen functionality, and, to date, no one has leveraged information describing peptide physiochemistry in genAI modeling. As such, there is an opportunity to leverage the physiochemical space inherent to neoantigens to generate diverse neoantigens that address sequence underrepresentation in the training data and recapitulate this information into precision cancer immunotherapy.

Here, we present ExoGAN, a genAI model that uses quantum chemistry feature engineering to integrate explainable chemical properties with sequence level data to design binding peptides with neoantigen functionalities. We demonstrate ExoGAN on human-specific MHC-I (HLA*A02:01) binding and experimentally validate a diverse selection of the resultant ExoGAN-generated peptides. For these selected peptides, we validated neoantigen activity at the recombinant protein level using biolayer interferometry and cellular scale using a professional antigen presentation assay. We show that ExoGAN-generated peptides are active TCR signalers for DMF5 in vitro, indicating that peptide physiochemistry features improve genAI model performance and enable more accurate neoantigen design. Next, we sought to deliver the ExoGAN-generated peptides to cancer cells. Current cancer immunotherapy treatment efficacy and patient response are still very low (<15%), which highlights the urgent needs of effective immunity modulation^29,30^. EVs have been recognized as the effective immunity regulators, useful in developing novel cancer immunotherapies^31^. Immune cell-derived EVs carry co-stimulatory factors and maintain presenting cellular origin by expressing surface MHC class I and class II molecules^32-34^, which could lead to highly effective anti-tumor immunity modulation. Indeed, our and other published research have observed that peptide/MHC binding complexes on EVs can be tailored and engineered for being capable of stimulating T cells^35,36^. Here, we incorporate a membrane surface molecular engineering approach we developed previously^35^ to fully decorate EVs with ExoGAN-generated peptides, experimental validation of which demonstrated effective T cell activation. Results from ExoGAN and EV-based immunity activation expand scientific understanding of neoantigen sequence diversity in the MHC-I space and advance cancer immunotherapeutic translational efforts through precision targeting and TCR activation. While we apply ExoGAN to human-specific MHC-I (HLA*A02:01) binding, the ExoGAN platform is generalized and can be applied to other binding targets. ExoGAN offers a generic strategy for precisely defining and developing EV surface peptide repertoires. It is the first pipeline to streamline MHC binding repertoires with T cell receptors via the EV delivery route, for developing a deployable and clinically translatable immunotherapy approach.

## Results

### ExoGAN Enables MHC-I Binding Peptide Prediction

**Figure 1:**
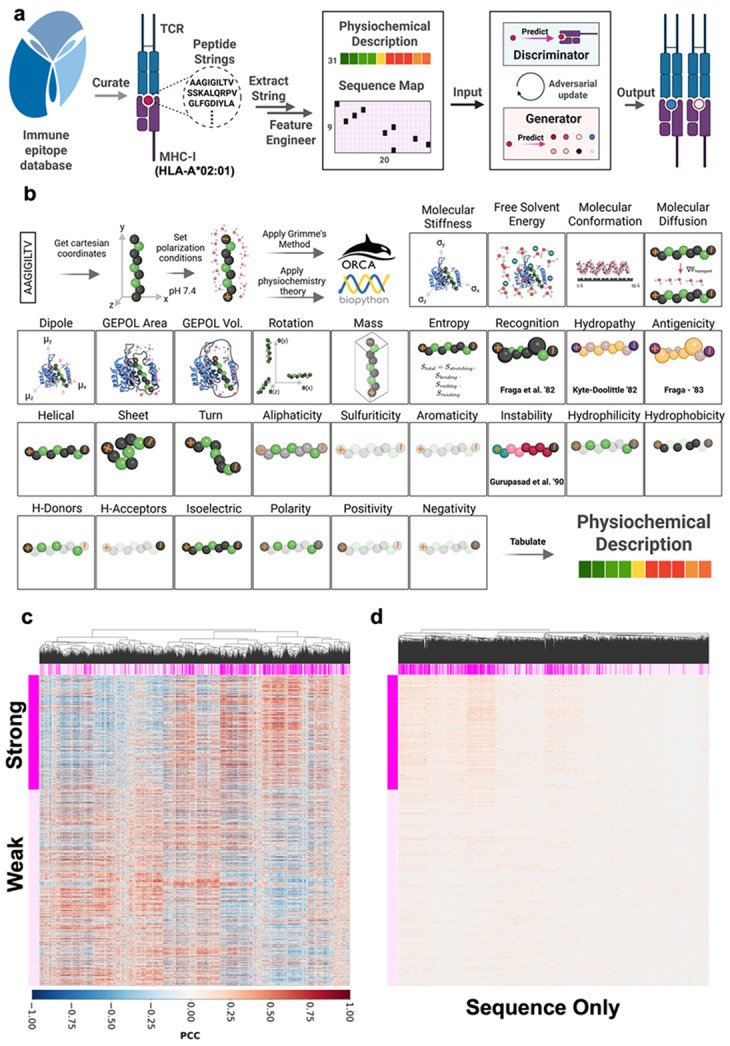
The ExoGAN genAI framework. (a) Schematic workflow of ExoGAN model. ExoGAN takes as input peptide sequences from IEDB that bind to MHC-I, then computes physiochemical features to generate the physiochemical molecular feature space. In this study, we showcased ExoGAN to develop peptides binding the most common MHC-I cancer allele, HLA-A*02:01. ExoGAN iteratively trains and computes new peptide sequences based on strong HLA-A*02:01 binding chemistry. **(b)** ExoGAN physiochemical feature engineering. Cartesian coordinates for each peptide sequence are calculated using Avogadro^42^, followed by changing the pH to 7.4, updating all amino acid chemistry, and setting polarization conditions to prepare each sequence for quantum chemical theory calculations by Grimme’s method ^43^ in the ORCA^24^ tool suite. Additional physiochemical theory was developed in Biopython^37^. (c) Heatmap showing pairwise correlation of the 7,860 IEDB peptides, calculated using the physiochemical features. Samples are grouped by binding affinity and, within each binding affinity group, hierarchical clustering was performed with Ward’s D2 method^44^ to assess HLA*A02:01 binding and inference based on antigen physiochemical clustering between the two groups. (d) Heatmap showing pairwise correlation of the 7,860 IEDB peptides based on sequence features. Samples are grouped by binding affinity and within each group, samples are sorted using hierarchical clustering.

ExoGAN is a generative framework leveraging quantum chemical feature engineering for explainable peptide design, implemented in Python and applied to neoantigen design and discovery (Fig 1a). In ExoGAN, peptide quantum chemical parameters (Fig 1b), indicating the required chemistry and buffer conditions to bind MHC-I, are computed using the ORCA toolkit^24^ and Biopython^37^ (**Supplementary Fig s1**). Each peptide’s quantum chemical feature map is then collated with their one-hot encoded peptide sequence map to form the combined ExoGAN feature space. ExoGAN utilizes a GAN architecture^38^ with a 1-D convolutional neural network topology for both generator and discriminator. At each iteration, ExoGAN generates new peptides by modifying the existing peptide sequences, then calls the ExoGAN functions to calculate quantum chemical parameter features for those new peptides. Using both sequence and quantum chemical features in training ensures that the generator is advancing into realistic peptide physiochemical spaces rather than relying solely on the chemistry of the training dataset. This iterative process is repeated until generator and discriminator’s loss gradients obtains a consecutive divergence (Δ_loss_ > 0.3, 5 epochs). Following peptide generation, we use phylogenetic analysis to identify a diverse set of ExoGAN-generated peptides that have the most overlapping physiochemistry with the training dataset, which we use to perform ranking by structural predictions and to identify a small set of peptides for computational and experimental validation. After identifying a set of peptides from phylogenetic analysis, we leveraged TCRModel2^4^ for the structural prediction of the MHC-I/TCR case. Detailed descriptions of the quantum chemical feature engineering and ExoGAN model implementation are listed in **Supplementary Note 1.1** and Methods, respectively.

We trained ExoGAN on IEDB data. Peptides were binned into strong and weak binders based on IEDB’s reported binding threshold^39^. We found that current sequence-only AI models may have been affected by limitations in the training dataset, and that residue bias may impact peptide binding predictions, such as NetMHCPan 4.1^7^ and CompariPSSM^40^. We sampled 1,000 conserved sequences using a Dirichlet prior for each strong and weak binding position weight matrices, fixing either leucine, valine, both, or neither. Next, we applied IEDB’s recommended peptide binding prediction model NetMHCPan 4.1 to each of these groups for comparison. We observed no statistical difference (p-adj <0.05, Bonferroni corrected Mann-Whitney-Wilcoxon) in NetMHCPan 4.1’s-predicted peptide affinity between the strong- and weak-binding peptide groups across any of the conditions (**Supplementary Fig s2**). We also utilized CompariPSSM on the strong and weak binding position scoring matrices and found similar residue preference across all the positions among strong and weak binder groups, and highly similar pocket importance for B (Gini = 92.5%) and F (Gini = 99%) (**Supplementary Fig s3**). CompariPSSM identified pockets B and F with biased residue positions but did not find statistical significance for leucine and valine preference (IWS adjust p-value = 0.85). As such, we determined that the IEDB data over-represents a subset of possible binding peptides.

To overcome this limitation in the training data, our ExoGAN model integrates peptide sequence data with a series of physiochemical features that we computationally derive from the sequence data, reducing the model’s reliance on sequence-level information and providing chemical and physical features which provide critical information about the molecular mechanisms driving peptide binding. The ExoGAN model calculates this information for each generated peptide and uses both sets of features for training. Thirty-one quantum chemical and physiochemical descriptors were utilized by ExoGAN to define HLA*A02:01 peptide binding chemistry (Fig 1b; described in Methods, **Supplementary Note 1.1**). We evaluated the physiochemical correlation in each class with known binding ânity by computing Fisher’s Z-transformation^41^ on the Pearson correlation coefficients (**Supplementary Fig s4**). We found that strong binding peptides have statistically significant preference (FDR < 0.05, **Supplementary Figs s5-7**) for sequences with more aromatic residues, molecular weight, diffusion, hydrophobicity, helical secondary structure, molecular volume and area, and dipole moment. Weak binding peptides prefer sequences with more polar neutral, aliphatic, and sulfur residues, faster molecular rotations, larger radius of gyration, increased hydrogen bond donation and hydrophilicity, consistent isoelectric points, and higher stiffness. Shared preferences (FDR > 0.05) between the groups included similar turn and sheet secondary structure, negativity and positivity, recognition factors, hydropathy, antigenicity, pocket B and F preference, entropy, hydrogen bond acceptors. Interestingly, the solvation energy contribution to binding affinity in the groups was almost statistically significant (FDR = 0.0709), suggesting that weak binding peptides may be more unstable. Next, we performed hierarchical clustering on the sample-by-sample correlation matrix in the feature engineered (Fig 1c) and sequence only spaces (Fig 1d). We also computed the silhouette and subsequent correlation matrix entropy scores to quantify physiochemical features and identify which features may be most effective for predicting binding affinity (**Supplementary Fig s8**). Peptide physiochemistry resulted in higher median silhouette scores (0.046 vs 0.009), sample silhouette distribution peaks (0.19 and 0.06), and lower entropy (2.389 vs 4.82) compared to sequence-only features. Thus, the ExoGAN physiochemistry feature engineering adds critical information relating to binding affinity and that likely enhances its ability to predict binding affinity compared to when solely using sequence features.

To further validate the effectiveness of physiochemical feature engineering to modeling peptide binding, we applied 4 explainable AI models (random forest, linear discriminant analysis, support vector machine, and logistic regression) to the ExoGAN training data to predict the binding affinity labels. We observed that all the models performed well regarding specificity, selectivity, F1, AUROC, recall, and Matthew’s Correlation Coefficient (MCC), when utilizing the physiochemical feature set (**Supplementary Table s1**). Given the importance of the physiochemical features, we leveraged Shapley Additive Explanations (SHAP)^45^ to find overlapping physiochemical importance across the explainable models (**Supplementary Figures s9-12**). All explainable models found total consensus for the top 9 most important physiochemistry: a peptide’s molecular rotation in the Z direction. Interestingly, the hydrophobic pocket window for HLA*A02:01’s B and F pockets may be the most informative predictors for binding affinity. Three of 4 models found that peptide’s molecular rotation in Y, dipole in X, and solvation energy, were informative predictors. Two of the 4 models found that peptide’s molecular volume, stiffness, radius of gyration, and entropy, were informative. The sum of importance of the remaining features was greater than any single top feature, which was listed for both the random forest (**Supplementary Figure s9**) and support vector machine (**Supplementary Figure s10**) models. Logistic regression (**Supplementary Figure s11**) and Linear discriminant analysis (**Supplementary Figure s12**) instead showed that the remaining feature sums were not as important for predicting binding affinity. Overall, the explainable models demonstrated that the engineered features used in ExoGAN training are effective descriptors of binding affinity and have the potential to boost ExoGAN performance.

To assess the impact of model architecture on performance in the ExoGAN framework, we tested simple frameworks including 1-D (ExoGAN’s discriminator) and 2-D convolutional neural networks, transformer, and long-term short memory models, training each architecture on the peptide sequence, physiochemistry, or combined feature maps. We observed substantial improvements in performance metrics for all models trained on physiochemical maps compared to those trained on sequence maps alone (balanced accuracy, F1, AUROC, MCC, specificity, and sensitivity; **Supplementary Table s3**). When trained on sequence information alone, ExoGAN’s discriminator generally outperformed all other models in performance metrics, including balanced accuracy, F1, AUROC, MCC, specificity, and sensitivity (**Supplementary Table s2**). When comparing models trained on both the sequence and physiochemical maps together, the 1-D CNN and the transformer architecture tied for top performance (Nemenyi-corrected Friedman’s test p-adj < 0.05, **Supplementary Table s4-5**). Lastly, we compared the ExoGAN discriminator’s ability to predict HLA*A02:01 binding affinity to state-of-the-art models, ACME^19^ and MHCFlurry^6^, and observed that the simple 1-D CNN used by ExoGAN outperform both (Wilcoxon corrected Kruskal-Wallis, p-adj < 0.05, **Supplementary Table s6**). This suggests that, rather than the architecture, the feature maps used by ExoGAN, which integrate domain knowledge into peptide generation and evaluation, are critical to its success.

### Leveraging ExoGAN to discover peptides with neoantigen potential

**Figure 2:**
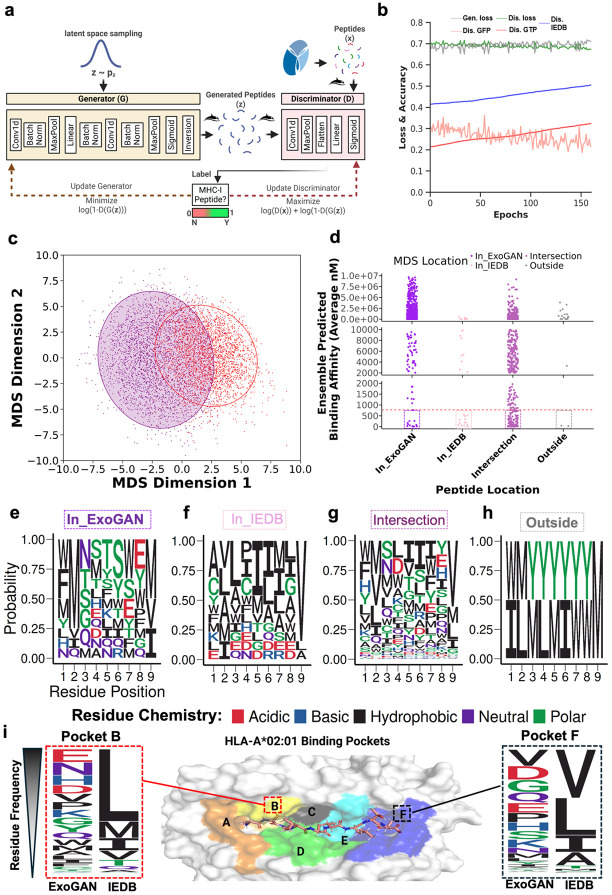
ExoGAN expands the HLA-A*02:01 sequence space by leveraging physiochemistry. (a) The ExoGAN model architecture. ExoGAN is a GAN, composed of two convolutional neural networks linked together. The generator samples from a latent space, operates, and determines amino acid probability per position. The inversion utilizes a one-hot encoding transformation to take the maximum probability of amino acid and determine the appropriate positional residue. The discriminator intakes the peptide feature map, including our physiochemical features and residue positions, and determines the chemical probability that peptides are either strong or weak binding HLA*A02:01 peptides. (b). ExoGAN training and performance over 160 iterations (1 month training time), visualizing the generator and discriminator’s performance and real and generated antigens. The figure also shows the ExoGAN discriminator’s performance on the 7,860 HLA*A02:01 peptides from IEDB to monitor performance in peptides with known strong and weak binding affinity. (c) A scatter plot showing multidimensional scaling on the physiochemical features between real and generated antigens. Ellipses are shown for the ExoGAN-generated peptides and those from IEDB. The ellipse angle was calculated as the covariance in multidimensional scaling space, while the ellipse radius represents a 95% confidence interval (d) Results from an ensemble of gold-standard models to predict HLA*A02:01 peptide affinity, applied to the ExoGAN-generated peptides, grouped by their MDS location. The ensemble (N=6) model prediction is shown. (e-h). Sequence logo plots showing peptide sequence for each of the MDS groups, to analyze residue bias. (i) Sequence logo plots depicting the residue frequency between IEDB and ExoGAN-generated peptides at HLA*A02:01’s b and f binding pockets, respectively.

ExoGAN takes as input both sequence and physiochemical features to model HLA*A02:01 binding and to output peptides with potential neoantigen functionality (Fig 2a). We trained ExoGAN for 160 iterations while monitoring the discriminator’s performance on the training dataset. ExoGAN reached convergence at 160 iterations, based on the generator and discriminator’s trending loss. Fig 2b shows improved ExoGAN classification performance in the IEDB dataset during out-of-distribution training, indicating that at each iteration of training, ExoGAN is better able to classify strong and weak binding peptides in the IEDB training data.

**Figure 3:**
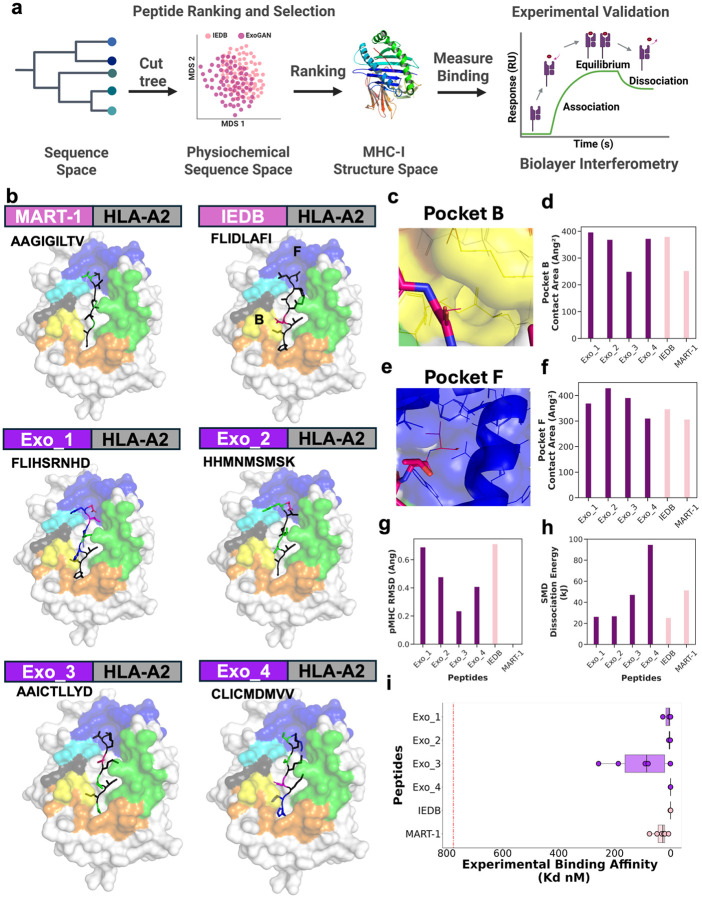
Selecting and evaluating a small set of sequentially-diverse ExoGAN peptides (a). We used phylogenetic analysis to define 28 clades of peptide sequences. These were filtered into groups based on the presence or absence of overlapping physiochemistry between ExoGAN and IEDB in each clade. One representative peptide from each clade was selected using PAM clustering to determine the most representative ExoGAN peptide sequence, which was then analyzed using structural prediction, computational ranking, and experimental validation of affinity to HLA*A02:01. Based on this analysis, 4 ExoGAN-generated peptides were selected for further experimentation. (b) We applied a pMHC-TCR prediction model, TCRModel2, to predict the interaction likelihood of this complex with the 4 representative ExoGAN peptides. Each peptide shown is presented by HLA-A*02:01, residues are colored by their chemical property, and HLA*A02:01’s binding pockets are lettered as described^53^. We evaluated the predicted pMHC structures using a series of metrics including the (c-d) pocket B contact area, (e-f) pocket F contact area, (g) pMHC RMSD, and (h) peptide dissociation with steering molecular dynamics. TCRModel2’s general performance metrics including prediction confidence, TCR-pMHC-IPTM, IPTM, and PLDDT scores are listed in Fig s17. (i) A 2:1 binding interaction model to compute the dissociation constant (Kd) as the binding affinity shows all sequences are high affinity for HLA*A02:01.

To investigate if the physiochemical space ExoGAN learns intersects with the physiochemical space within the IEDB dataset, we reduced dimensionality on the collated physiochemistry with multidimensional scaling (MDS) and computed a 95% confidence ellipse for each group (Fig 2c). MDS visualization showed 4 physiochemical areas for the generated peptides: one area that is exclusive to ExoGAN peptides, another exclusive to IEDB peptides, a third area encompassing the intersection of ExoGAN and IEDB peptides, and a four area of peptides that fell outside the 95% confidence ellipse. Despite overlapping physiochemistry within these 4 areas, average sequence similarity across the areas indicates high levels of sequence diversity regardless of area membership (Fig 2c). To evaluate if current gold-standard HLA*A02:01 prediction models latently capture sequence physiochemistry in their predictions, we created an ensemble of gold-standard models listed by IEDB, including ANN 4.0, NetMHCPan 4.1, SMM, and SMMPBMEC, and ACME^19^ and MHCFlurry^6^ (Ensemble). This Ensemble model was applied to ExoGAN-generated peptide sequences, grouped by each sequence’s location in the MDS plot. We found that the Ensemble prediction underestimated the expected binding affinity across all the locations (Fig 2d). Within sequences that the Ensemble model predicted as high affinity, we observed significant hydrophobic residue bias for pockets B and F (Fig 2e-h), corresponding with hydrophobic residue bias frequency in the IEDB training data and our earlier analysis with CompariPSSM. While the residue frequency analysis of ExoGAN’s peptides revealed that ExoGAN is capturing the hydrophobic residue frequency in pocket B and F (Fig 2i), there is a significant shift in the residue distribution leading to a difference in the apparent peptide bias presented to the Ensemble. To determine whether this residue bias is ExoGAN identifying binding peptides that are under-represented in the training data or if this is ExoGAN inaccurately generating binding peptides, we performed additional computational analysis of the peptides and selected a diverse set for in-depth experimental validation (Fig 3).

### Selecting sequentially-diverse ExoGAN peptides and validating their binding affinity

**Figure 4:**
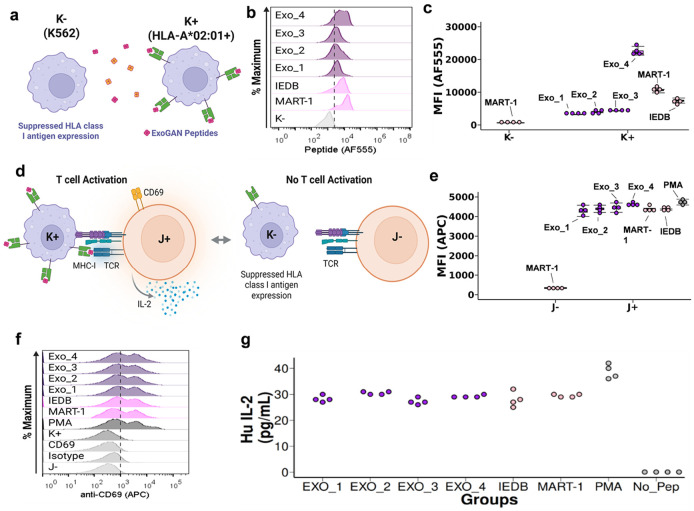
Biological validation of ExoGAN-generated peptides. (a) Visualization of the flow cytometry assay indicating how HLA*A02:01 antigen affinity on engineered K562 cells (K+) was determined, using a similar method to Saini et al^19^. **(b) Results from the flow cytometry assay.** Cytogram distributions of AF555 fluorescent intensity on each cell per sample group. The K− group received MART-1, while all other groups used K+ cells. (c) Quantification of the HLA*A02:01 sequence cytogram. The median fluorescent intensity (MFI) across all groups was computed (N=4), indicating all sequences are bound to HLA*A02:01. **(d) Visualization of the DMF5 signaling assay.** Once K+ cells were observed to present ExoGAN-generated peptides, K+ cells presenting the peptides were subjected to interact with engineered Jurkats exogenously expressing the DMF5 TCR allele (J+) for 24 hours. We then assessed neoantigen potential by measuring J+’s CD69 abundance and IL-2 secretion. J− cells do not express DMF5. **(e) Cytogram assessment of DMF5 TCR signaling.** Cytogram distributions showing CD69’s fluorescent intensity (APC) per sample group. **(f)** The median fluorescent intensity of CD69 across J+ and J− groups was computed (N=4), indicating that all sequences elicit DMF5 signaling. (g) An IL-2 enzyme-linked immunosorbent assay (ELISA) was performed to measure J+’s IL-2 secretion after 24 hours (N=4).

We aimed to find the most representative peptides for further computational analysis and experimental validation of their capability to bind HLA*A02:01 (Fig 4a). Given that overlapping physiochemical properties enable diverse peptide sequences to adopt similar functional and binding capabilities, we constructed a phylogenetic tree containing all ExoGAN and IEDB sequences using unweighted pair-group means arithmetic under a raw differences model (**Supplementary Figure s13a**, see Methods). This phylogeny was used to identify sequence-level clusters within and across the IEDB and ExoGAN data, based on sequence-level similarities. Clusters were defined as isolated clades after cutting the phylogeny for a branch length of 4.10 (**Supplementary Figure s13b**). For each cluster, we quantified group similarity by calculating the average Hamming distance of each pair of peptides within one cluster versus across clusters, to determine average cluster similarity. We found high sequence diversity regardless of group membership **(Supplementary Figure s14 and 15)**. However, when we assessed if ExoGAN and IEDB physiochemistry overlapped in a sequence-level cluster with MDS (**Supplementary Figure s16**), we confirmed that 20 of the 28 clusters indicated overlap (**Supplementary Figure s17**). Within each of these 20 clusters with overlapping IEDB and ExoGAN physiochemistry, we identified the most representative ExoGAN sequence in the cluster using partition around medoid (PAM) clustering. The most representative ExoGAN-generated peptide from each cluster was then added to a peptide library to be considered for additional experimental validation (**Table s7**).

We next computationally evaluating neoantigen potential for each peptide in the peptide library described above by leveraging TCRModel2 (an HLA-TCR structure prediction model) against known HLA*A02:01 tumor associated antigens. These tumor-associated antigens were manually curated by the Cancer Antigenic Peptide Database (CAPED)^46^. TCRModel2 requires a known TCR for the prediction and we chose the DMF5 allele^47-49^ as our representative TCR since ExoGAN’s training data included MART-1 (27-35; *AAGIGILTV),* a known melanoma tumor associated antigen.Results from TCRModel2 indicate that ExoGAN-generated peptides are structurally equivalent to HLA*A02:01’s known tumor-associated antigens (two-sided Wilcoxon-Mann-Whitney, p > 0.05; **Supplementary Figure s18**). Based on TCRModel2’s performance, we selected 4 ExoGAN-generated peptides and 2 controls to experimentally validate. We chose 3 top- and 1 lower-ranked ExoGAN-generated sequences, 1 top-ranked sequence from IEDB, and MART-1 to predict binding with the Ensemble model used previously. These sequences are *FLIHSRNHD* (Exo_1), *HHMNMSMSK* (Exo_2), *AAICTLLYD* (Exo_3), *CLICMDMVV* (Exo_4), *FLIDLAFLI* (IEDB), and *AAGIGILTV* (MART-1). The top ranked sequences were Exo_4 (93% confidence), Exo_3 (92%) and Exo_2 (91%), while Exo_1 (88%) was the lower ranked sequence. IEDB and MART-1 were predicted at 91% and 93%, respectively. These 6 sequences are tested experimentally.

Analysis of results from the Ensemble model (described above) indicated limitations in the existing gold standard binding affinity prediction models; These models tend to predict strong binding in peptides with hydrophobic residues in the anchor positions (**Supplementary Figure s19**) despite each sequence representing their cluster’s physiochemistry. Even our biological positive control, MART-1, was predicted as a weak binder, conflicting with MART-1’s known affinity^47-51^. However, prediction by TCRModel2 shows that the peptide binding structure for all generated sequences match well with MART-1 (Fig 3b). TCRModel2’s fitting coefficients for three ranked models, including the IPTM, the TCR-pMHC IPTM, and confidence showed no statistical significance (p > 0.05, One-way ANOVA, **Supplementary Figure s20**).

Focusing on HLA*A02:01 anchor pockets B (Fig 3c) and F (Fig 3e), we determined that ExoGAN-generated sequences have a higher contact area in these pockets when compared to MART-1 (Fig 3d and 3f). We computed each sequence’s pMHC RMSD to MART-1, showing that all structures are less than 1 angstrom, indicating strong interaction with HLA*A02:01 (Fig 3g). To verify the structural prediction analysis by TCRModel2, we conducted steering molecular dynamics (SMD)^52^, a computational chemistry strategy that calculates the energy to dissociate the peptide from HLA*A02:01 5 nanometers away (see Methods). Weaker binding peptides will have significantly lower energy to leave HLA*A02:01’s pocket and diffuse into the solvent. In this design, we used polyGly and the dipeptide, GL, as our baseline negative controls to observe that a threshold of 7 kJ indicates weak binding (**Table s8**). Our SMD analysis indicated that all 6 tested peptides (4 ExoGAN, 1 IEDB, and 1 MART-1) strongly bind to HLA*A02:01, requiring a minimum of 25 kJ to leave the pocket (Fig 3h **and Supplementary Video s1**). Moreover, SMD analysis revealed that Exo_4, Exo_3 and MART-1 were significantly stronger to bind HLA*A02:01 than all other peptides, while Exo_1 and 2 were comparable to IEDB. Finally, we synthesized these peptides using solid-phase synthesis and controlled for quality by HPLC-MS (see Methods; **Supplementary Figures s21-27**) and performed experimental validation of sequence affinity with biolayer interferometry (Fig 3i **and Supplementary Figure s28**). All sequences displayed their own transport kinetics to interact, bind, and refold inside of HLA*A02:01. Further, when we computed the binding affinity using the 2-to-1 receptor-ligand interaction kinetic model^15-17^, all sequences demonstrated high affinity for HLA*A02:01 (Fig 3i).

### Experimental validation of neoantigen potential from ExoGAN peptides

Given ExoGAN-generated peptides were observed to bind HLA*A02:01, we performed a series of in vitro antigen presentation assays to determine neoantigen potential of the 6 peptides (4 ExoGAN, 1 IEDB, and 1 MART-1) selected above. To measure antigen presentation, we performed a similar method reported by Saini et al^19^ (Fig 4a). Saini et al.’s method exchanges a native peptide presented by MHC for a target peptide by incubating the target peptide with antigen presenting cells. The stronger binding target peptide will then diffuse and chemically exchange with the native peptide, which MHC presents for downstream antigen presentation. In our design, we utilized K562 cells that do not express MHC (K−) and engineered K562s^51,53^ (K+) that only expresses the HLA*A02:01 allele (see Methods). Following the protocol in Saini et al, we first demonstrated our K+/K− cell antigen presentation system by exchanging MART-1 onto HLA*A02:01. We showed that K+ cells presented MART-1 while K− cells did not present MART-1 (**Supplementary Fig s29**). Next, we used our K+/K− cell system to evaluate how ExoGAN-generated peptides exchanged and bound HLA*A02:01 by incubating these peptides with the K+ and K− cells. As in the biolayer interferometry results, we observed that K+ cells had comparable presentation of ExoGAN peptides as to MART-1 (Fig 4b). The IEDB and Exo_4 peptides were observed as the most potent peptide binders (Fig 4c), congruent with the Ensemble model predictions for their predicted affinity to HLA*A02:01. Next, we evaluated if ExoGAN peptides have neoantigen potential by performing a TCR signaling assay (Fig 4d). Based on DMF5’s responsivity to peptide presentation by K+ cells, in this assay we used engineered Jurkats, T cells that exogenously express the DMF5 TCR (J+), standardized against non-engineered Jurkats, T cells that do not express the DMF5 TCR (J−), to measure CD69 expression and IL-2 secretion. Our TCR signaling assay demonstrated that all generated sequences elicited comparable CD69 expression levels to MART-1 (Fig 4e and 4f), and IL-2 secretion (Fig 4g).

### ExoGAN enables EV-based immunotherapy

**Figure 5:**
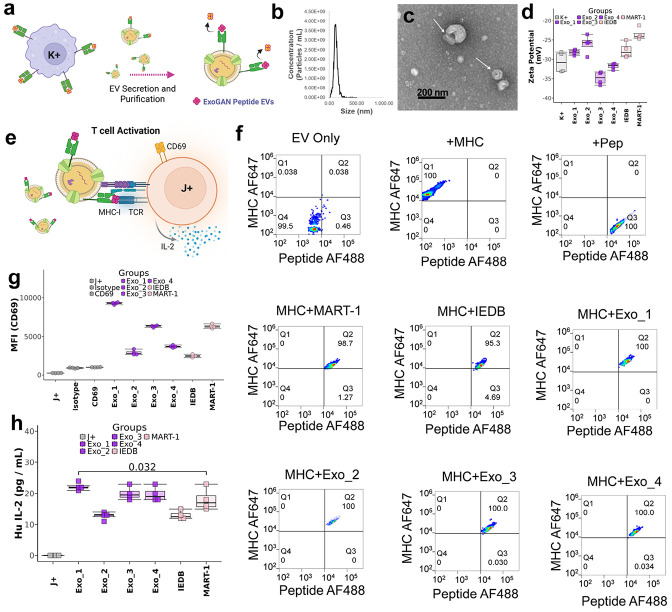
In vitro demonstration of EV precision cancer immunotherapy. (a) A visualization showing how antigen-presenting cell-derived EVs (APC-EVs) were isolated from K+ cells using EXODUS, followed by quality assessment with nanoparticle tracking analysis, transmission electron microscopy, and zeta potential. (b) Average size distribution for APC-EVs isolated by EXODUS. (c) A representative electron microscopy image shows APC-EVs isolated by EXODUS to indicate their size, distribution, and morphology. (d) APC-EVs pre-and-post sequence engineering were evaluated for their change in surface chemistry (N=4) to evaluate APC-EV potential. (e) A visualization of the DMF5 TCR signaling assay. Similar to the K+ cell presentation assay, ExoGAN engineered APC-EVs were subjected to present their peptides to activate J+ cells. We evaluated J+ cell activation by measuring CD69 expression and IL-2 secretion 24 hours using flow cytometry and the IL-2 ELISA, respectively. (f) Similar to the method reported by Saini et al., we performed nano-flow cytometry to measure peptide presentation on engineered APC-EVs. In this assay, anti-HLA-A*02:01 and fluorescent peptide were incubated with APC-EVs, unless specified, to evaluate the peptide binding to HLA-A*02:01. (g) The median fluorescent intensity of CD69 across all groups (N=4), indicating that ExoGAN peptide engineered APC-EVs elicit DMF5 signaling. (h) IL-2 secretion was quantified by ELISA after engineered APC-EVs were applied directly to J+ cells.

One major challenge in cancer immunotherapy is delivering the appropriate neoantigen to CD8+ T cells across the lymphatic system for differentiation into tumor-infiltrating lymphocytes. ExoGAN designs peptides, of which our experimental tests demonstrate several to be potential neoantigens. The next major step towards creating an ExoGAN-generated peptide immunotherapy is delivering these neoantigens to the cells. To showcase how ExoGAN may accelerate precision cancer immunotherapy, we leveraged antigen-presenting cell-derived EVs (APC-EVs), antigen cell-derived nanoparticles that carry MHC-I, and co-stimulatory factors that enable neoantigen delivery to CD8+ T cells. We designed EVs derived from K+ cells with ExoGAN-designed peptides to precisely activate J+ cells expressing the melanoma TCR, DMF5. (Fig 5a). First, we isolated EVs from K+ cells using EXODUS^54^ and found that isolated particle distribution, concentration (Fig 5b), and morphology (Fig 5c) were consistent with EV field standards^55^. Next, we re-evaluated if ExoGAN-generated peptides were binding, this time to the EV’s HLA*A2:01 by measuring the zeta potential and percentage of peptide exchange through nano flow cytometry. The zeta potential of EVs designed with all peptide groups showed a significant change in surface chemistry after the peptide exchange (Fig 5d). Confirming previous results, our nano flow cytometry assay showed that all ExoGAN-generated peptides reached 100% peptide binding to HLA*A02:01, while MART-1 and IEDB were 98.7% and 95.3% (Fig 5f). Using the IEDB peptide as our control to present the same number of peptides, we standardized the designed EV dose for all groups at 2x10^9^ particles to stimulate J+ cells (Fig 5e) and measured CD69 expression and IL-2 secretion. Through measuring the CD69’s median ûorescent intensity from J+ cells, we observed that Exo_1 outperformed MART-1, while Exo_2-Exo_4 were comparable to MART-1 and the IEDB peptide (Fig 5g). We also observed that the J+ cells secreted significantly more IL-2 for Exo_1 than MART-1 (Dunnett’s test corrected one-way ANOVA, p <0.05), while the other ExoGAN-generated EV groups were comparable to MART-1 (Fig 5h). Overall, the ExoGAN-designed EVs precisely activated J+ cells expressing the melanoma TCR, DMF5.

## Discussion

Here we present ExoGAN, a genAI framework which designs peptides for MHC presentation with downstream applications in precision immunotherapy (Fig 1). ExoGAN incorporates physiochemical feature engineering using QM/MM methods, which we demonstrated are more effective at determining how and why peptides bind to HLA-A*02:01. We show that these physiochemical features contained substantial information such that simple AI models trained on the physiochemical features outperformed state-of-the-art AI models trained on sequence-only data. All model architecture performed best when trained on both sequence and physiochemical features. Additionally, the inclusion of these features enabled the ExoGAN framework to overcome a significant training data limitation (Fig 2) by advancing toward a functional physiochemical probability space to infer significant peptide sequence variety that strongly bind HLA-A*02:01. While ExoGAN-generated peptides’ binding affinities were underpredicted by an ensemble of gold-standard MHC-I binding affinity models (Fig 3), we demonstrated both computationally and experimentally that ExoGAN-designed peptides are diverse, strong MHC-I binding peptides (Fig 4) that demonstrate specific T cell activation. This and previous works ^5,12,13,20^ suggest that existing binding affinity prediction AI models overfit to the IEDB training data. Additionally, although the ExoGAN framework was not trained to model T cell activation, we showed at both the peptide binding (Fig 5) and extracellular vesicle peptide presentation to elicit immunotherapeutic T cell activation (**Fig 6**); that the ExoGAN-designed peptides were either outperforming (Exo_1) or comparable (Exo_2-4) to the known melanoma tumor-associated antigen (MART-1) in activating a melanoma-specific TCR, DMF5, on T cells.

Previous genAI models for peptide design have relied on peptide sequence information and sequence-to-protein structural interaction. In the context of designing peptides binding HLA-A*02:01, we showed that the strong and weak binding peptides for HLA-A*02:01 are indistinguishable from each other (**Supplementary Figs s2-3**), suggesting that current state-of-the-art genAI models may be overfitting to learn small differences in sequence-feature frequency rather than sequence binding function. We posited that a latent physiochemical space, calculated from the sequence information and presented to ExoGAN, provides domain-specific context of the molecular rules of binding affinity in a way that AI models do not innately capture, and that inclusion of these features improves binding affinity prediction and novel peptide generation. We addressed this problem in genAI modeling by incorporating physiochemical information from QM/MM methods into a genAI framework, ExoGAN. This is the first report in literature to combine QM/MM with genAI. ExoGAN integrates all this information plus the sequence-level data during model training and evaluation. For HLA-A*02:01, we applied ExoGAN to 2,895 strong binding peptides from the IEDB and generated 2,895 novel peptides. An additional 4,965 weak binding peptides from IEDB were used to evaluate changes in ExoGAN model performance during training and to compare to other AI models. We found that, while sequence-level data may be informative to group strong and weak HLA-A*02:01 binding antigens (Fig 1d **and Supplementary Figure s8b**), the physiochemical features provided additional information that may explain the apparent grouping (Fig 1c, **Supplementary Figure s4-8a**), indicating the urgency for new methods that correct sequence-biased models predicting antigen binding affinity to HLA-A*02:01, and broadly, MHC-I. Explainable AI feature analysis using SHAP enabled us to probe the features driving changes in model performance that are not available to most sequence-only models trained on deep learning architectures **(Supplementary Table s1 and Figures s9-12)** and these simpler models required fewer parameters and compute resources. Next, we showed how various- and-simple ExoGAN discriminator architectures (**Supplementary Tables s2-4**) trained using physiochemical features described here outperformed the state-of-the-art sequence only models (**Supplementary Table s6**). Since physiochemistry directly probes peptide function, our models likely have strong predictive power when HLA-A*02:01 binding despite significantly less model complexity and parameters. Given these discoveries, we hypothesized that generative models utilizing our physiochemical features and sequence information to generate new peptide sequences are more likely to be pushed into a significantly more representative sequence functional space based on inferable physiochemistry.

We validated our hypothesis that physiochemical information is likely required to generate functional MHC-I neoantigens by successfully generating diverse sequences with a GAN (Fig 2a). The GAN functions by recapitulating out-of-distribution neoantigen sequences against the IEDB training dataset; however, given the data sparsity and likely non-representative sequence set in the training data, we expected ExoGAN to suffer from mode collapse and vanishing gradients. We instead observed that ExoGAN was converging and performed increasingly well on classifying binding affinity in the IEDB dataset (7,865 peptides total, 2,895 strong and 4,965 weak binders; blue line in Fig 2b). This suggests that learning using sequence physiochemistry likely enables AI models to learn the full distribution space of sequence family functionality, even in the scenario where training data is not representative of the full sample distribution space. We posited that, if the sequences binding HLA-A*02:01 have similar physiochemistry, then significant sequence diversity is allowable. We showed that ExoGAN inferred the known antigen physiochemistry (Fig 2c) to expand into new sequence feature spaces. Thus, our results suggest that our inclusion of physiochemical descriptors may enhance all sequence-based models to address the in-sequence distribution bias to correct for the out-of-distribution sequence bias. Given that that overall physiochemistry is likely similar between sequences despite distinctly different sequence-level features, we hypothesized that the current sequence-only models would underpredict the binding affinity of ExoGAN-generated sequences. Indeed, this hypothesis was directly proven since an ensemble of gold-standard MHC-I binding models underpredicted binding in ExoGAN-generated peptides (Fig 2d-h). By extension, current genAI frameworks ^5,12,13,20^ have been shown to produce highly similar sequences to the training dataset. ExoGAN captured greater sequence diversity of HLA-A*02:01 binding functionality by using physiochemistry-enhanced sequence maps in addition to peptide sequence (Fig 2i)

It is critical to have diverse MHC binding peptides for a given immunotherapeutic target because patient-specific genetic variations give rise to therapeutic inconsistency. Current binding affinity models are biased towards a subset of binding peptide sequences. We and others^21-23^ demonstrate this through computational and experimental analysis, and external reports using these models have shown functional validation of such predicted sequences. ExoGAN is the only QM/MM framework to-date that directly infers MHC-I binding, and we show here that ExoGAN generates multiple sequence families (**Supplementary Figures s13-s16**) with overlapping physiochemistry (**Supplementary Figure s17)** whose binding affinity is incorrectly predicted by the existing sequence-only AI models. Of the 4 diverse ExoGAN-generated peptides we experimentally tested, only one (Exo_4; *CLICMDMVV)* was predicted correctly by the gold-standard model ensemble, while three other ExoGAN peptides (Exo_1-Exo_3; *FLIHSRNHD, HHMNMSMSK, AAICTLLYD*) and a known tumor-associated antigen, MART-1 (*AAGIGILTV*) were not **(Supplementary Figure s19)**. Structural prediction (Fig 3b-g, **Supplementary Figure s18**) and experimental demonstration via biolayer interferometry indicated that all 4 of the ExoGAN neoantigens have high binding affinity for HLA*A02:01 (Fig 3h). Biolayer interferometry provides information on a peptide’s mass transport, MHC-I binding, and subsequent refolding inside MHC-I (**Supplementary Figure s27**). Using biolayer interferometry, we reported the first experimental method to measure interactions of single peptide binding events to MHC-I. Our results showed that peptides binding MHC-I is highly dependent on peptide physiochemistry, which ExoGAN infers as part of peptide generation and binding affinity prediction. These results corroborated our central hypothesis that physiochemical modelling in a genAI framework captures MHC-I functionality, and more broadly, that physiochemical features correct known issues in existing sequence-only models, to better understand inconsistences in modelling peptide binding affinity.

HLA’s polymorphic distribution^56,57^ considerably increases the difficulty in predicting peptide binding specificity to MHC and subsequent presentation to TCRs. To test this, we tested immunologic activity in 6 peptides (4 ExoGAN-generated peptides, a peptide from IEDB, and a known control) using K562 (K−) and K562 cells expressing only HLA-A*02:01 (K+) rigorously investigate if ExoGAN-generated antigens are immunologically active (**Supplementary Figure s28**). Through the K−/K+ cell system (Fig 4a), we mitigated HLA’s polymorphic bias to verify ExoGAN’s capability to model MHC presentation. Given that ExoGAN-generated neoantigens were experimentally confirmed to bind HLA*A02:01 in this cell system (Fig 4b-c), we sought to control the expected TCR polymorphism that creates considerable variability in patient immunoresponse. Thus, we can rigorously test ExoGAN’s latent ability to design peptides that stimulate an immune response. Through the J−/J+ cell system (Fig 4d), we showed that all 4 of ExoGAN-generated neoantigens induce immunostimulation (Fig 4e-g). We further corroborated these cellular results using extracellular vesicle associated peptide presentation approach for developing precision immunotherapy (Fig 5). EVs from K+ cells carry HLA-A*02:01 which can be replaced and decorated with stronger binding ExoGAN peptides. Results from this study showed that one ExoGAN peptide (Exo_1) outperformed the melanoma associated antigen MART-1 (Fig 5i) and the other 3 ExoGAN-generated peptides showed comparable T cell immunoactivation.

Binding affinity is one crucial factor in designing neoantigens. ExoGAN peptide design covers broader effective antigens which could advance mechanistic understanding of neoantigen functionality, which is critical given the inconsistent TCR signaling across human patients. We demonstrated computationally and experimentally that the ExoGAN-generated peptides bind and stimulate the T cell activation for eliciting immune response. ExoGAN may also accelerate precision cancer immunotherapy development by using EVs which demonstrated the direct activation of J+ cells expressing the melanoma TCR, DMF5. The result indicates the potential for EV-delivered ExoGAN-designed peptides. Overall, the increased scientific understanding of neoantigen diversity across tumors, discovered by ExoGAN, will also accelerate the development of new functional immunotherapy agents in activating patient-specific TCRs.

## Methods

### ExoGAN Framework

ExoGAN uses a GAN model^38^, composed of two linked convolutional neural networks (a generator and discriminator) as illustrated in Fig 2, plus sequence and biophysiochemical features to generate novel peptides that bind to HLA*A02:01. ExoGAN is a generalizable model and can accept any set of peptide samples. In the application used in this manuscript, the ExoGAN generator takes input of a 2,895 values vector, one per strong binding peptide from IEDB. The generator’s input is then passed into a 1D convolutional layer with filter size 3, then normalized, max pooled, then applied onto a fully connected linear layer to sample amino acid spatial relations, followed by normalization, 1d convolution, normalized and pooled, then evaluated by a sigmoid activation to output amino acid likelihood and subsequent peptide sequence. The generator output will be a list of peptide sequences (2,895 x 1, in the application used here) for the discriminator’s evaluation. The discriminator’s input is one-hot encoded peptide sequences collated with their physiochemical descriptors (size = 51x9). The discriminator’s input is then passed into a 1d convolutional layer with filter size 2, normalized, max pooled, flattened, then applied onto a fully connected linear layer, followed by sigmoidal activation to evaluate the peptide’s probability of HLA-A*02:01 binding.

### ExoGAN’s dataset selection and physiochemical feature engineering

HLA*A02:01 peptides were downloaded from the Immunological Epitope Database (IEDB)^2^ on March 18, 2023. IEDB is a public repository listing HLA alleles containing information about binding affinity. IEDB uses a classification threshold of ≥0.486 and ≤0.362 to designate strong binding and weak binding peptides within each HLA allele. After obtaining all IEDB HLA*A02:01 sequences and parsing for strong and weak binding sequences, we applied feature engineering for each HLA*A02:01 using ORCA^24^ and Biopython^37^. ORCA is an open-source software suite capable of applying quantum chemical theory to optimize peptide structures and obtain electron-geometry dependent properties. For high-throughput generation of ORCA command scripts, we implemented functions using Avogadro^42^, applying a quick pre-optimization using AMBER. Given lengthy computation by density functional theories (DFT), we opted for the semi-empirical calculation with HF-3c^43^, reported to be comparable to DFT for small peptides and molecules under KDIIS optimization^58^. We also applied our calculations on rigid backbone structures. To set the molecular environment, we applied the Conductor-like Polarizable Continuum model (CPCM) in universal solvation mode ^59,60^ with a water box sized 50 at pH 7.4. After the HF-3c calculation was complete, we wrote functions using Biopython to calculate additional peptide features for ExoGAN. After all physiochemical features were calculated for all peptides, we applied scikit-learn’s^61^ RobustScaler to standardize the feature scales across peptides. A list of all features and their descriptions are listed within **Supplementary Note 1.1**

### ExoGAN’s training procedure

To train ExoGAN, we utilized a 1:1 learning rate (2 x 10^−5^) and equal training steps (N=160) for the generator and discriminator, using 3 batches, each containing 1,930 peptide sequences. We used the Adaptive momentum^62^ (Adam) learning scheme to optimize the networks. While also monitoring ExoGAN’s losses and generated sequence classification, we assessed ExoGAN by evaluating the discriminator’s performance on 7,860 peptides from IEDB, including the 2,895 strong binding peptides used to train the model and an additional 4,965 weak binding peptides. For every iteration, generated sequences were feature engineered by ORCA and Biopython, where each sequence required 15 AMD EPYC CPUs with 10 GB memory, with the average compute time per sequence under 10 minutes. The total time to run ExoGAN was 710 hrs (29.5 days).

### CompariPSSM

To evaluate the strong and weak binding position weight matrices in the IEDB training set for binding motifs, we utilized the CompariPSSM webserver (https://slim.icr.ac.uk/projects/comparipssm). CompariPSSM is a tool that derives motif determinants by comparing the background probability of seeing similar residues by chance to a calculated importance weight similarity (IWS) and dissimilarity (IWD) score between two position weight matrices.

### Fisher’s Z transformation and subsequent two-sided Z test

To evaluate the statistical correlations each physiochemical feature has on peptide binding affinity, we applied Fisher’s Z transformation. Fisher’s Z transformation transforms Pearson correlation coefficients into Z distributed values amenable to a two-sided Z-based hypothesis test evaluating if the correlations observed between two features are statistically significant using the following formula:

(1) $$Z_{test}=\frac{arctanh(z_{1})-arctanh(z_{2})}{\sqrt{\frac{1}{z_{1_{k}}-3}+\frac{1}{z_{2_{k}}-3}}}$$

### SHAP Analysis of physiochemical feature importance using explainable models

To evaluate how informative the physiochemical features were to distinguish strong vs weak HLA*A02:01 binding sequences, a model survey including logistic regression, random forest, linear discriminant analysis, and support vector machine were utilized. A grid search using StratifiedKFold^61^ (N=10) to maximize the balanced accuracy was performed to find the best parameters for each model, followed by deploying the parameters to generate an optimized model using StratifiedKFold (N=10). The folds were evaluated by the MCC, F1 Score, Balanced Accuracy, Specificity, Precision, and Recall. A list of model parameters used is listed in **Supplementary Note 1.2.** Next, SHAP analysis was performed on the optimized models.

### Architectural evaluation on feature maps and comparison to published HLA*A02:01 binding models

A simple 1-D and 2-D CNN, transformer, and LSTM architecture were used in the ExoGAN architecture. For each feature map, where the feature map was sequence, physiochemistry, or sequence plus physiochemistry, we trained and evaluated each architecture using the same StratifiedKFold splits from the SHAP analysis, evaluating the MCC, F1 Score, Balanced Accuracy, Specificity, Precision, and Recall. The Friedman rank-sum aggregate test with Nemenyi’s post-hoc test was performed to investigate architectural differences in performance between the feature maps. All models were used to compare with recently published and experimentally validated HLA*A02:01 prediction models, MHCFlurry and ACME.

### External model performance on ExoGAN-generated peptide sequences

To evaluate if ExoGAN’s generated sequences were outside IEDB’s probability distribution and subsequent implicit data bias, we leveraged gold-standard, experimentally validated HLA*A02:01 binding models from IEDB including ANN, SMPMBEC, NetMHCPan, SMM, as well as two experimentally validated deep learning models, MHCFlurry 2.0 and ACME. All models purely leverage sequence homology to evaluate HLA*A02:01 binding prediction. IEDB’s model description and their parameters are listed in **Supplementary Note 1.3.**

### Phylogeny construction, analysis, and ranking of generated sequences

To construct the phylogeny between ExoGAN and IEDB’s strong binding sequence dataset, we leveraged the Molecular Evolutionary Genetics Analysis^63^ (MEGA11) software to use the Unweighted Pair-Group Method with Arithmetic Mean^64^ (UPGMA) with raw-differences model for phylogeny construction. We applied Felsenstein’s bootstrap method^65^ (N=50) to ensure the constructed phylogeny’s topology had a 95% confidence. To visualize and condense the phylogeny based on branch length, we utilized the Interactive Tree of Life (iTOL)^66^. After condensing the phylogeny, we extracted the peptide sequences within each node to assess sequence similarity by calculating the average hamming distance for each sequence within each node. Following, we computed each node’s physiochemical space through principal component analysis, followed by filtering out nodes where there was no spatial overlap of generated sequences with IEDB’s sequences. Nodes with molecular overlap were subjected to Partition Around Medoid^67^ (PAM) clustering on their average hamming distance, visualized through multidimensional scaling. Within each node, the most central generated and IEDB sequence was determined by the closest generated and IEDB sequence to the medoid. All node’s central sequences were tabulated and then ranked by TCRModel2^4^, a structural prediction algorithm to predict HLA*A02:01 antigen presentation to the DMF5 allele of the target T cell receptor. After ranking the generated sequences, the top 3 and 1 apparent sub-par generated sequence were selected for synthesis and downstream validation of HLA*A2:01 affinity.

### TCRModel2 and structural prediction analysis

TCRModel2 is an AlphaFoldv2.3-specific framework that generates high resolution models of TCR-pMHC complexes (https://github.com/piercelab/tcrmodel2). TCRModel2 uses a reduced multiple sequence alignment database such that only prospective TCR and MHC hits were available across the Small Big Fantastic Database, UniRef90, Uniprot, and PDB. TCRModel2 will output prediction metrics like AlphaFoldv2.3 including predicted local difference distance test (pLDDT), predicted topological modelling (pTM), interfacial pTM (ipTM), and the model confidence (0.2 x pTM + 0.8 x ipTM). We computed additional metrics including the Pocket B and F contact area, describing the all-atom interaction between MHC-I and peptide residues in the 2^nd^ and 9^th^ positions, using a cutoff of <3.5 Å, and computing the residues surface area. Additionally, we computed the pMHC root mean square deviation (RMSD) between pMHC structures, and the peptide dissociation energy using steering molecular dynamics (SMD).

### Steering Molecular Dynamics (SMD)

SMD is a computational technique to apply a constant force to a biomolecular to measure the work (energy) required to translate specific distances using the following formula:

(2) $$F_{dissociation}=\sum_{i=0}^{N}\frac{1}{2}k_{spring}{\left(z_{t_{i}}-z_{t_{i+1}}\right)}^{2}$$

This approach was applied through the OpenMM framework^68^. Specifically, we used TCRModel2 predicted structures, cleaned up PDB errors using PDBFixer and set the molecular environment to pH 7.4. The random seed was set to 4247357. Next, we applied an Amber14 force field using the tip3pfb water box, Particle Mesh Ewald as our electrostatics methods, and heavy water molecules. For the pulling velocity, we used 0.0005 nanometers/picosecond with a spring constant of 150 kj/mol*nm. The integration used was The LangevinMiddleIntegrator at 300 K, friction coefficient at 1/ps, and 4 femtoseconds per integration step using a constraint tolerance of 0.0001. We prepared the simulation by first minimizing the energy for 100 steps, then gradually equilibrated the temperature to 300 K in 100 steps, then gradually equilibrated to a pressure of 1 atm in 100 steps. We calculated the rate of change in the peptide’s Y center of mass as the pulling force was applied until the peptide sequence was 5 nm away. To compute the dissociation energy, the following formula was applied:

(3) $$E_{dissociation}=\sum_{i=0}^{N}k_{spring}(z_{t_{i}}-z_{t_{i+1}})$$

### Peptide synthesis and quality control

Chemical regents were purchases from Aapptec, Sigma Aldrich, CEM and TCI America and used without further purification. Solid phase peptide synthesis was performed using a CEM Liberty Blue Automated Microwave Peptide Synthesizer utilizing standard Fmoc chemistry with preloaded Wang resin. Single coupling reactions were employed at each coupling using standard microwave conditions (90 0C, 2 min). A solution of 20% piperidine in DMF was utilized for Fmoc deprotection reactions. The activating reagent of 0.5 M DIC in DMF and activator base of 1 M Oxyma in DMF, was used for amino acid couplings. Once the synthesis was completed the resin was washed four times with DCM and cleaved by agitating in a solution of 92.5:2.5:2.5:2.5 TFA: TIPS: H2O: DODt for 2 h. Purification of the peptides were carried out by reverse phase preparative HPLC on Agilent 1260 series preparative pumps using a C18 column (5 mm particle size, 19 mm x 150 mm), with a gradient of water/ acetonitrile (90:10 to 0:100 containing 0.1% TFA, flow rate 19 mL/min)) over 40 min. Fractions containing the desired peptide were combined and lyophilized to give a white powder. Analytical HPLC traces were acquired using an Agilent 1100 quaternary pump and a Hamilton PRP-1 (polystyrene-divinylbenzene) reverse phase analytical column (7 μm particle size, 4 mm x 25 cm) with UV detection at 210 nm. The elution was achieved with gradients of water/ acetonitrile (90:10 to 0:100 containing 0.1% TFA, flow rate 1 mL/min) over 20 min. LRMS (ESI+) was acquired on a SHIMADZU Single Quadrupole mass spectrometer. Peaks are reported as m/z.

### Biolayer interferometry of neoantigen affinity to recombinant HLA*A2:01

Biolayer interferometry^69^ is an optical technique for measuring macromolecular interactions by analyzing interference patterns of white light reflected from the surface of the biosensor, determining kinetics and affinity of molecular interactions through measuring the shift in the interference pattern when the concentration of target molecules is changed upon the event. Biotinylated HLA-A*02:01 presenting the GL dipeptide is first bound to the streptavidin coated biosensor, then sequentially washed in wells containing wash buffer to remove excess HLA-A*02:01. Next, the biosensor is subjected to a well containing the generated sequence to measure binding affinity to HLA-A*02:01 by replacing the bound peptide over 3000 seconds to measure the association rate constant, K_on_. After, the biosensor tip is subjected to another well containing wash buffer for 3000 seconds to measure the dissociation rate constant, K_off_.The binding affinity, K_d_ (nM), is computed by the ratio K_off_ / K_on._ A range of peptide concentrations was used to generate multiple curves in properly selecting the right curve fitting parameters and calculation of HLA-A*02:01 binding affinity.

### *Biological validation of neoantigen affinity to surface* HLA-A*02:01 *and subsequent pMHC functionality*

To validate ExoGAN at the cellular level, we used engineered K562^53^ cells (K+) that only express surface HLA*A02:01. Native K562 cells (K−) and K+ cells were grown in complete 1640-RPMI (Gibco) media and harvested at 1 x 10^6^ cells. Based on the method reported by Saini et al.^70^, cells were incubated with 1 uM AF555 N-terminal tagged peptides with or without the 5 uM GL dipeptide in 1 uM brefeldin A for 3 hours in a 96 well plate. Total volume was 200 uL per well. Cells were washed 3x and subjected to flow cytometry for HLA*A02:01 affinity. To investigate neoantigen functionality, we loaded 1 x 10^5^ K+ cells with 1 uM peptides for 3 hours. Next, we added 2 x 10^5^ engineered Jurkat cells that present the DMF5 T cell receptor allele (J+) to each well. After 24 hours, CD69 and IL-2 was measured by flow cytometry and ELISA, respectively to evaluate neoantigen functionality. For flow cytometry, 1% BSA and FC block (bio-rad) was combined with all cell solutions for 30 minutes before antibody staining and washing. J− cells were used as a negative control where necessary.

### Extracellular vesicle isolation by EXODUS

K+ cell media was harvested every 3 days to a volume of 30 mL. The media was then differentially centrifuged at 2000x RCF, supernatant was aspirated to a new tube, then 10000x RCF for 15 minutes at 4°C for aspiration to a new tube. This aspirated solution was filtered through a 0.22 micron polyethylsulfone filter before EV isolation by EXODUS. EXODUS’s manufacturer instructions were followed to isolate EVs into a 200 uL solution of 1x PBS with 25 mM trehalose. 100 uL was used for nanoparticle tracking analysis and transmission electron microscopy.

### Nanoparticle Tracking Analysis

Nanoparticle tracking analysis was conducted using ZetaView (QUATT, Particle Metrix Inc, USA). ZetaView measures the nanoparticle’s Brownian motion using an incident laser to determine the corresponding size. The nanoparticle motion is then tracked by the detector and recorded over time: The incident laser wavelength was 488 nm^−1^ with sensitivity at 75 and shutter time at 163, over 90 seconds at the highest video resolution for all 11 positions.

### Transmission electron microscopy

Transmission electron microscopy (TEM, FEI Spirit TEM 120 kV) verified the morphology of isolated EVs. Briefly, ultrathin copper grids coated with 400 mesh carbon film (FCF400-Cu-UB, Electron Microscopy Science, USA) were used with glow discharge treatment for 1 min before use. Then, 5 μl EV samples were individually added onto the glow-discharged grids and were quiescent for 10 min at room temperature. The grids were washed with distilled water once, then negatively stained with filtered 2% aqueous uranyl acetate for 2 min and dried at room temperature before observation. The TEM imaging power was 120 kV by FEI Spirit G2 with a digital camera (Soft Image System, Morada and Gatan Orius SC 1000B CCD-camera).

### K+ extracellular vesicle peptide engineering and J+ cell immunostimulation

100 uL of the isolate solution was combined with a 100 uL mastermix solution containing 5 uM GL dipeptide and 1 uM peptide. The 200 uL solution was incubated on a revolver at 4°c for 1.5 hours, following purification by ultrafiltration using a 10k MWCO column into a 30 uL solution. 10 uL was used for zeta potential, nano flow cytometry to assess peptides binding onto HLA-A*02:01 and 10 uL was used to assess CD69 expression and IL-2 secretion from J+ cells. To measure J+ functionality, 2x10^5^ J+ cells in 190 uL were added to a 96 round bottom well plate, followed by adding the 10 uL isolate solution. 24 hours later, CD69 was evaluated with flow cytometry

### Zeta Potential

Zeta potential was measured using the Litesizer 500 (Anton Paar, Austria). The Litesizer 500 measures the zeta potential by flowing an electric current between two electrodes within the cuvette, measuring the interfacial charge of the solution at the particle’s surface and correlating this to the particle’s surface charge. 5 uL of EV isolates with different peptides were dispersed into a 0.1x PBS solution at pH 7.4 for measurement.

### Nanoflow Cytometry

NanoFCM (NanoFCM, Inc., Maryland, USA) was used to assess peptide binding onto HLA-A*02:01 on K+ EV isolate membranes. Similar to the assay by Saini et al, 100 uL of 1 uM AF647-tagged peptides with 5 uM GL dipeptide was combined with 100 uL of EV isolates. The 200 uL solution was purified by ultrafiltration with a 10k MWCO column by filtering down to 50 uL using 4000 xRCF at 4°c for 10 minutes, adding 200 uL ice-cold 1x PBS, then repeating again for two 2 washes. The 50 uL was then recovered by inverting the column and spinning at 1000 xRCF for 1 minute. To stain for HLA*A02:01, this 50 uL was incubated with FC block (bio-rad) and 1% BSA to a total volume of 150 uL for 30 minutes at 4°c. After, 10 uL of a 1:1000x diluted HLA-A*02:01 antibody tagged with AF488 was incubated with this solution to a total volume of 160 uL. Following NanoFCM’s manufacturer instructions, a 1:100 dilution of the final solution was made in 1% BSA and FCblock for the nano flow cytometry analysis.

## Supplementary Material

The online version contains supplementary material available at [XXXX].

This is a list of supplementary files associated with this preprint. Click to download.

GA.jpeg

20250617EXOGANPEPTIDEDATASET.csv

Supplementaryvideo1.mp4

20250617ExoGANSupplementaryInformation.pdf
